# Organometallic Complex Strongly Impairs Chikungunya Virus Entry to the Host Cells

**DOI:** 10.3389/fmicb.2020.608924

**Published:** 2020-12-15

**Authors:** Débora Moraes de Oliveira, Igor de Andrade Santos, Daniel Oliveira Silva Martins, Yasmim Garcia Gonçalves, Léia Cardoso-Sousa, Robinson Sabino-Silva, Gustavo Von Poelhsitz, Eduardo de Faria Franca, Nilson Nicolau-Junior, Carolina Colombelli Pacca, Andres Merits, Mark Harris, Ana Carolina Gomes Jardim

**Affiliations:** ^1^Institute of Biomedical Science, Federal University of Uberlândia, Uberlândia, Brazil; ^2^Institute of Bioscience, Language and Exact Sciences, São Paulo State University, São José do Rio Preto, Brazil; ^3^Institute of Chemistry, Federal University of Uberlândia, Uberlândia, Brazil; ^4^Institute of Biotechnology, Federal University of Uberlândia, Uberlândia, Brazil; ^5^FACERES Medical School, São José do Rio Preto, Brazil; ^6^Institute of Technology, University of Tartu, Tartu, Estonia; ^7^Faculty of Biological Sciences and Astbury Centre for Structural Molecular Biology, University of Leeds, Leeds, United Kingdom

**Keywords:** antiviral, arboviruses, Chikungunya virus, ruthenium α-Phellandrene, organometallic complex

## Abstract

Chikungunya fever is a disease caused by the Chikungunya virus (CHIKV) that is transmitted by the bite of the female of *Aedes* sp. mosquito. The symptoms include fever, muscle aches, skin rash, and severe joint pains. The disease may develop into a chronic condition and joint pain for months or years. Currently, there is no effective antiviral treatment against CHIKV infection. Treatments based on natural compounds have been widely studied, as many drugs were produced by using natural molecules and their derivatives. Alpha-phellandrene (α-Phe) is a naturally occurring organic compound that is a ligand for ruthenium, forming the organometallic complex [Ru_2_Cl_4_(p-cymene)_2_] (RcP). Organometallic complexes have shown promising as candidate molecules to a new generation of compounds that presented relevant biological properties, however, there is a lack of knowledge concerning the anti-CHIKV activity of these complexes. The present work evaluated the effects of the RcP and its precursors, the hydrate ruthenium(III) chloride salt (RuCl_3_⋅xH_2_O) (Ru) and α-Phe, on CHIKV infection *in vitro*. To this, BHK-21 cells were infected with CHIKV-*nanoluciferase* (CHIKV*-nanoluc*), a viral construct harboring the *nanoluciferase* reporter gene, at the presence or absence of the compounds for 16 h. Cytotoxicity and impact on infectivity were analyzed. The results demonstrated that RcP exhibited a strong therapeutic potential judged by the selective index > 40. Antiviral effects of RcP on different stages of the CHIKV replicative cycle were investigated; the results showed that it affected early stages of virus infection reducing virus replication by 77% at non-cytotoxic concentrations. Further assays demonstrated the virucidal activity of the compound that completely blocked virus infectivity. *In silico* molecular docking calculations suggested different binding interactions between aromatic rings of RcP and the loop of amino acids of the E2 envelope CHIKV glycoprotein mainly through hydrophobic interactions. Additionally, infrared spectroscopy spectral analysis indicated interactions of RcP with CHIKV glycoproteins. These data suggest that RcP may act on CHIKV particles, disrupting virus entry to the host cells. Therefore, RcP may represent a strong candidate for the development of anti-CHIKV drugs.

## Introduction

The Chikungunya virus (CHIKV) belongs to the genus *Alphavirus* of the family *Togaviridae* (Chen et al., 2018; ICTV, 2019). It is a positive strand RNA virus with a genome of approximately 12 kb (Schuffenecker et al., 2006). The icosahedral capsid is covered by a lipid envelope derived from the host cell membrane and contains viral glycoproteins E1 and E2 (Khan et al., 2002; Schuffenecker et al., 2006; Thiberville et al., 2013).

CHIKV is transmitted through the bite of the female mosquito of *Aedes* sp. (Vu et al., 2017) and is the causative agent of Chikungunya fever being related to epidemics mainly in tropical and subtropical regions (Khan et al., 2002; Paixão et al., 2018; Stegmann-Planchard et al., 2019). CHIKV was first isolated during an epidemic in Tanzania in 1953 (Robinsson, 1955; Wintachai et al., 2012). In 2006, CHIKV outbreaks were reported on several Indian Ocean islands and resulted in about 250 deaths on the French island of *La Réunion* (Schuffenecker et al., 2006). In 2013, the virus was detected in the Americas with reported cases in the Caribbean islands (Kaur and Chu, 2013). The first case in Brazil was reported in 2014 (Carvalho et al., 2014).

Chikungunya fever presents symptoms as fever, prostration, muscle aches, lymphopenia, and arthralgia (Cunha et al., 2017; Paixão et al., 2018). Pain associated to arthralgia in the phalanges, wrists, and ankles occurs in up to 98% of cases (Thiberville et al., 2013). The infection can progress to a chronic condition in around 70% of infected patients (de Andrade et al., 2010; Simon et al., 2011). Muscle pain and persistent arthralgia may last for periods ranging from months to years (Mathew et al., 2017).

Currently, there is no vaccine or specific therapy against CHIKV infection (Yang et al., 2017; Dey et al., 2019). Treatment of symptomatic CHIKV infections is palliative, based on the use of non-salicylate analgesics and non-steroidal anti-inflammatory drugs (Parashar and Cherian, 2014; Mathew et al., 2017).

Several of the currently used drugs for different pathologies are either from natural origin or synthesized based on natural scaffolds (Teixeira et al., 2014; da Silva-Júnior et al., 2017). Alpha-phellandrene (α-Ph) is an organic compound of the monoterpene class (Zhang et al., 2008), which has already been shown to have antimicrobial, antifungal, anti-cancer, and anti-diabetic properties (Inouye et al., 2001; Elshafie et al., 2016; Zhang et al., 2017; Cox-Georgian et al., 2019). It is a common ligand for ruthenium (Bennett et al., 2007), a metal belonging to the iron group, that has shown to possess effective biological properties as antimicrobial when complexed to other molecules (Pavan et al., 2010). The antitumoral activity of the ruthenium α-Phellandrene complex [Ru_2_Cl_4_(p-cymene)_2_] (RcP) has also been described (Clarke et al., 1999; Habtemariam et al., 2006; Dougan and Sadler, 2007; Dyson, 2007; Vajs et al., 2015; Savić et al., 2020), however, to the best of our knowledge, the antiviral properties of the RcP have not been assessed yet.

Here we evaluated the antiviral activity of the RcP and its precursors, the hydrate ruthenium(III) chloride salt (RuCl_3_⋅xH_2_O) (Ru) and the α-Ph, on the CHIKV replicative cycle. These data are the first description of the anti-CHIKV activities of RcP complex.

## Materials and Methods

### Compounds

**FIGURE 1:**
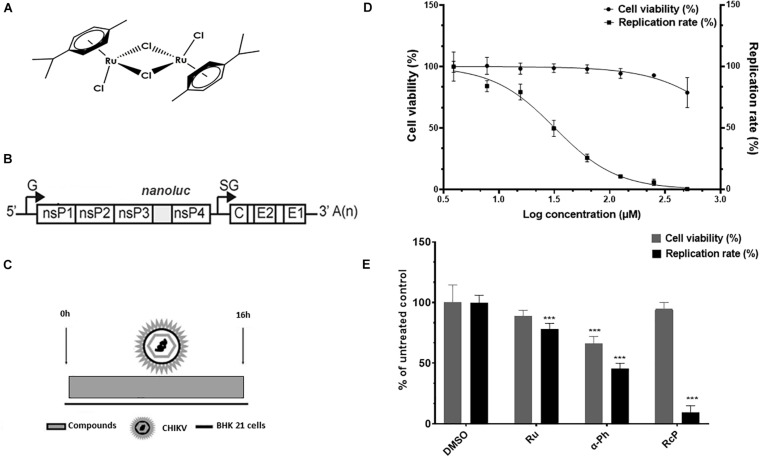
CHIKV activity of ruthenium α-phellandrene complex (RcP). **(A)** RcP chemical structure. **(B)** Schematic representation of CHIKV-*nanoluc* genome. **(C)** Schematic representation of infectivity assays. **(D)** Cells were treated with concentrations of RcP ranging from 3.9 to 500 μM and the effective concentration of 50% (EC_50_) and cytotoxic concentration of 50% (CC_50_) were determined. CHIKV replication was measured by *nanoluciferase* assay (indicated by □) and cellular viability measured using an MTT assay (indicated by •). Mean values of three independent experiments each measured in quadruplicate including the standard deviation are shown. **(E)** BHK-21 cells were infected with CHIKV-*nanoluc* at MOI 0.1 and treated with compounds at 125 μM for 16 h. Virus infectivity and cell viability assays were performed. ***P < 0.01 was considered significant.

The ruthenium and α-Phellandrene complex ([Ru_2_Cl_4_(p-cymene)_2_], RcP) (Figure 1A) was synthesized as previously described (Jensen et al., 1998). The hydrate ruthenium salt (RuCl_3_.xH_2_O) (Ru) and alpha-phellandrene (α-Ph) used in the synthesis of complex were purchased from Sigma-Aldrich. The complex was dissolved in dimethyl sulfoxide (DMSO), a coordinating solvent, and stored at −20°C in order to decrease the kinetic exchange of chloride and ligands. The stock solution was stored in aliquots that were thawed only once. Dilutions of the compounds were made immediately prior to the experiments. For all the assays performed, control cells were treated with media containing DMSO at the final concentration of 0.3%.

### Cell Culture

BHK-21 cells (fibroblasts derived from Syrian golden hamster kidney; ATCC CCL-10) were maintained in Dulbecco’s modified Eagle’s media (DMEM; Sigma-Aldrich) supplemented with 100 U/mL of penicillin (Hyclone Laboratories, United States), 100 mg/mL of streptomycin (Hyclone Laboratories, United States), 1% dilution of the stock of non-essential amino acids (Hyclone Laboratories, United States), and 1% of fetal bovine serum (FBS; Hyclone Laboratories, United States) in a humidified 5% CO_2_ incubator at 37°C.

### Virus

The CHIKV-*nanoluciferase* (CHIKV*-nanoluc*) (Figure 1B; Matkovic et al., 2018) used for the antiviral assays was based on CHIKV LR2006-OPY1 strain (East/Central/South African genotype). The cDNA plasmid of CMV-CHIKV-*nanoluc* contains CMV promoter, SV40 terminator and a sequence encoding *nanoluciferase* protein inserted into the region encoding the C-terminal domain of viral nsP3 protein (Pohjala et al., 2011; Matkovic et al., 2018). For virus rescue, 2.3 × 10^7^ BHK-21 cells seeded in a T175 cm^2^ flask were transfected with 1.5 μg of CMV-CHIKV-*nanoluc* plasmid, using lipofectamine 3000^®^ and Opti-Mem media. Forty-eight hours post transfection the supernatant containing CHIKV-*nanoluc* particles was collected, clarified by filtration and stored at −80°C. To determine viral titer, 5 × 10^5^ BHK-21 cells were seeded in each well of a 6 well plate 24 h prior to the infection. Then, the cells were infected with 10-fold serially dilutions of CHIKV*-nanoluc* for 1 h at 37°C. The inocula were removed and the cells were washed with PBS to remove the unbound virus and incubated with cell culture media supplemented with 1% penicillin, 1% streptomycin, 2% FBS, and 1% carboxymethyl cellulose (CMC). Infected cells were incubated for 2 days in a humidified 5% CO_2_ incubator at 37°C, followed by fixation with 4% formaldehyde and stained with 0.5% violet crystal. The viral plaques were counted to determine CHIKV*-nanoluc* titer (Sabine, 1993).

### Cell Viability Through MTT Assay

Cell viability was measured by MTT [3-(4,5-dimethylthiazol-2-yl)-2,5-diphenyl tetrazolium bromide] (Sigma-Aldrich) assay. BHK-21 cells at the density of 5 × 10^4^ were cultured in each well of 48 well plates and treated with different concentrations of each compound at 37°C with 5% of CO_2_; DMSO was used as vehicle control. 16 h post treatment, compound-containing media was removed and MTT solution at 1 mg/mL was added to each well, incubated for 1 h and replaced with 100 μL of DMSO (dimethyl sulfoxide) to solubilize the formazan crystals. The absorbance was measured at 560 nm on Glomax microplate reader (Promega). Cell viability was calculated according to the equation (T/C) × 100%, in which T and C represented the optical density of the treated well and control groups, respectively. The cytotoxic concentration of 50% (CC_50_) was calculated using Graph Pad Prism 5.0 Software.

### Antiviral Assays

To assess the antiviral activity of compounds, BHK-21 cells were seeded at density of 5 × 10^4^ cells per well into 48 well plates 24 h prior to the infection with CHIKV-*nanoluc* at a multiplicity of infection (MOI) of 0.1 plaque forming unit/cell. Compounds were simultaneously added to cells. Samples were harvested in Renilla luciferase lysis buffer (Promega) at 16 h post-infection (h.p.i.) and virus replication levels were quantified by measuring *nanoluciferase* activity using the Renilla luciferase Assay System (Promega). The effective concentration of 50% inhibition (EC_50_) was calculated using Graph Pad Prism 5.0 Software. The values of CC_50_ and EC_50_ were used to calculate the selectivity index (SI = CC_50_/EC_50_).

To investigate which step of CHIKV replicative cycle was targeted by the compound, BHK-21 cells at the density of 5 × 10^4^ cells per well were seeded in a 48 well plate 24 h prior to infection and/or treatment for all assays. For all experiments conducted to evaluate the effects of the compound on different stages of replicative cycle, MOI used to infect cells was 0.1 and replication rates were assessed measuring luminescence levels at 16 h.p.i. To evaluate if the compound possesses protective activity to the host cells, cells were treated for 1 h with the compound before infection, extensively washed with PBS (phosphate buffered saline) to remove compound and then infected with CHIKV*-nanoluc*. The effect on the entry steps was analyzed by incubating virus and compound simultaneously with BHK-21 cells for 1 h. Then, cells were washed for the removal of the inoculum and replaced by fresh media. To investigate the activity of the compound on post-entry stages of viral replicative cycle, cells were infected with CHIKV for 1 h, washed extensively with PBS to remove unbound virus after which media containing compound was added.

The virucidal activity of the compound was assessed by previously incubating virus and compound for 1 h at 37°C and then adding the inoculum to the cells for an extra 1 h. The inoculum was removed and replaced by fresh media. To evaluate the attachment step, the cells were treated with virus and compound for 1 h at 4°C, and then were washed with PBS and replaced by media. For the uncoating step, virus and compound were incubated with cells for 1 h at 4°C followed by 30 min at 37°C, and then washed and replaced by media.

### Quantum Mechanics and Molecular Docking Studies

The RcP molecular structure was optimized using the ORCA 4.2.1 (Neese, 2018) program. B3LYP density functional model (comprising Slater local exchange, the Becke non-local exchange, the Vosco-Wilk-Nusair local correlation functional and the Lee-Yang-Parr non-local correlation functional) along with basis set LANL2DZ (Los Alamos National Laboratory 2-double-ζ), which also incorporates an “Effective Core Potential,” were used for calculations (Cao and Ren, 2011). Absence of negative frequencies in vibrational analysis of complexes confirmed that the optimized geometry was the lowest energy structure. Docking studies were performed using autodock4.2 (Morris et al., 2009) with RcP ligand. The crystal structure of the mature envelope glycoprotein complex (furin cleavage) of CHIKV used as target protein (PDB code: 3N42) was obtained from the Protein Data Bank. The dock procedures were performed using the AutoDockTools 1.5.6 interface along with autogrid4 and autodock4 (AD4). To prepare the ligand to molecular docking, the AutoDockTools 1.5.6 (Sanner, 1999) added Gasteiger charges and converted the target crystal structure in a format required for AD4. The Lamarckian genetic algorithm in Autodock were: population (150 individuals); maximum energy evaluations (25,000,000); maximum generations (27,000); one individual surviving into next generation; genetic algorithm docking runs (150); and a ranked cluster analysis was performed using an RMS tolerance of 2.0 Å on each docking calculation. The grid map used was 118 × 112 × 110 points, centers of grid: *x* = −18.369; *y* = 13.506; *z* = −22.667 with a grid space of 0.375 Å. Ligand interactions were analyzed using the Discovery Studio Visualizer program (BIOVIA, 2020).

### Infrared Spectroscopy Spectral Data Analysis

A micro-attenuated total reflectance (ATR) device coupled to an ATR-FTIR spectrophotometer Vertex 70 (Bruker Optics, Reinstetten, Germany) was used to record the infrared fingerprint between 1,800 cm^–1^ and 400 cm^–1^region. The internal-reflection element of ATR unit is blended by a diamond disk. The infrared beam is reflected at the interface toward the sample in the ATR-crystal. The background of the air spectrum was removed in all ATR-FTIR dataset. One microliter of each sample was dried using airflow on ATR-crystal for 2.5 min. Then, sample spectra were recorded in triplicate. All samples were acquired with 4 cm^–1^ of resolution and 32 scans were performed to each sample analysis. The mean spectra were normalized by the vector method and adjusted to rubber band baseline correction. The original data were plotted in the Origin Pro 9.0 (OriginLab, Northampton, MA, United States) software to perform the second derivative analysis, which was obtained by applying Savitzky-Golay algorithm with polynomial order 5 and 20 points of the window. The height of valley indicated the intensity of functional group evaluated (Dinache et al., 2020).

### Statistical Analysis

Individual experiments were performed in triplicate and all assays were performed a minimum of three times in order to confirm the reproducibility of the results. Differences between means of readings were compared using analysis of variance (one-way or two-way ANOVA) or Student’s *t*-test using Graph Pad Prism 5.0 software (Graph Pad Software). *P*-values < 0.01 were considered to be statistically significant.

## Results

### Ruthenium and Alpha-Phellandrene Complex (RcP) Inhibits CHIKV Infection *in vitro*

Since ruthenium complexes were shown to possess antimicrobial properties (Pavan et al., 2010), we aimed to investigate the anti-CHIKV activity of the RcP (Figure 1A) by using a recombinant CHIKV that expresses the *nanoluciferase* reporter (CHIKV-*nanoluc*) (Figure 1B).

To assess the effect of RcP on cell viability and virus infection, we therefore performed a dose response assay to determine the effective concentration of 50% (EC_50_) and the cytotoxicity concentration of 50% (CC_50_) values for RcP. BHK-21 cells were infected with CHIKV-*nanoluc* and treated with RcP at concentrations ranging from 3.9 to 500 μM and viral replication efficiency was evaluated measuring activities of *nanoluciferase* reporter at 16 h.p.i. (Figure 1C). In parallel, cell viability was measured by MTT assay. The results showed that RcP was able to completely block the virus replication while the minimum cell viability was 84% of that of DMSO treated control cells (Figure 1D). By the use of this range of concentrations, it was determined that the RcP complex has an EC_50_ of 32 μM, CC_50_ > 1,300 μM and Selective Index (SI) of 43.

Since the complex was shown to be strongly active against CHIKV at a concentration of 125 μM with no cytotoxicity, we also evaluated effects of its precursors, the hydrate ruthenium salt RuCl_3_.xH_2_O (Ru) and the α-Phellandrene (α-Ph), at this concentration on cell viability and viral replication. Cells were infected with CHIKV-*nanoluc* at the MOI of 0.1 and treated with RcP, Ru or α-Ph at 125 μM. The efficiency of viral replication and cell viability were evaluated after 16 h by luminescence and MTT assays, respectively (Figure 1C). The results showed that RcP significantly (*p* < 0.01) inhibited 91% of CHIKV replication and presented no toxicity to cells (Figure 1E). Albeit, Ru and α-Ph decreased cell viability and/or presented reduced antiviral activity compared to the complex (*p* < 0.01) (Figure 1E). This data demonstrated that RcP exhibited the best therapeutic index (favorable ratio of cytotoxicity to antiviral potency) and was therefore selected for further analysis.

### RcP Strongly Inhibits CHIKV Entry to the Host Cells

The antiviral activity of the RcP at 125 μM on different stages of CHIKV replication was analyzed by infecting cells with CHIKV*-nanoluc* at the MOI of 0.1 and measuring luminescence levels 16 h.p.i., for all performed assays.

**FIGURE 2:**
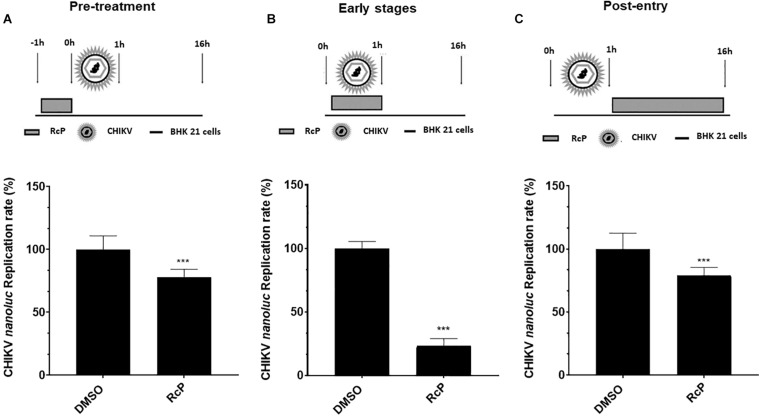
Time of addition analysis reveals that RcP mainly inhibits entry step of CHIKV infection. **(A)** Schematic presentation and results of pre-treatment experiment. **(B)** Schematic presentation and results of experiment analyzing effect of RcP on early stages of infection. **(C)** Schematic presentation and results of post-treatment experiment. For all assays, CHIKV replication was analyzed by measuring of *nanoluc* activity at 16 h.p.i. Mean values of a minimum of three independent experiments each measured in triplicate. ***P < 0.01 was considered significant.

First, BHK-21 cells were pretreated with RcP for 1 h at 37°C, washed with PBS to completely remove the compound and then were infected with CHIKV*-nanoluc* (Figure 2A). In this experiment, the RcP demonstrated a modest yet significant (*p* < 0.01) reduction of 23% of luminescence levels (Figure 2A).

To evaluate the effect of RcP on the early stages of infection, virus and RcP were simultaneously added to BHK-21 cells, treatment lasted for 1 h, then washed with PBS and was followed by incubation in the absence of compound (Figure 2B). Under these conditions, treatment with RcP significantly (*p* < 0.01) and prominently (by ∼77%) reduced virus replication indicating that RcP inhibits virus entry to the host cells (Figure 2B).

In order to reveal whether RcP also inhibited post-entry steps, the cells were first infected with CHIKV*-nanoluc* for 1 h at 37°C, washed to remove unbound virus and then incubated with compound containing media (Figure 2C). RcP also demonstrated a modest yet significant (*p* < 0.01) reduction of 21% of luminescence levels when the treatment was performed after virus entry to the cells (Figure 2C). However, it cannot be excluded that RcP inhibited spread of progeny virus from infected cells to non-infected cells instead of post-entry stages. Altogether, our data clearly suggest that the predominant antiviral activity of RcP is related to its ability to inhibit the entry stage of the virus lifecycle. Therefore, the activity of RcP on CHIKV entry to the cells was further evaluated.

**FIGURE 3:**
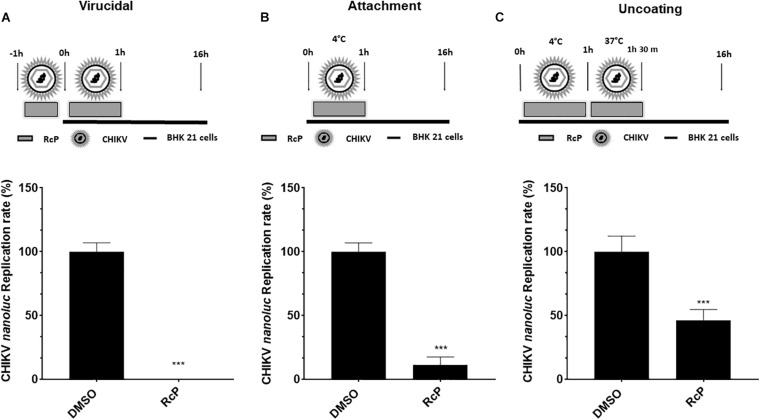
RcP exhibits strong virucidal activity. **(A)** Schematic presentation and results of virucidal assay. **(B)** Schematic presentation and results of attachment inhibition assay. **(C)** Schematic presentation and results of uncoating inhibition assay. For all assays, CHIKV replication was measured by *nanoluc* activity at 16 h.p.i. Mean values of a minimum of three independent experiments each measured in triplicate ***P < 0.01 was considered significant.

First, supernatant containing CHIKV-*nanoluc* was incubated with RcP for 1 h at 37°C, prior to the infection of cells, to investigate if the compound possesses a virucidal effect. The inoculum of virus and RcP was transferred to naïve BHK-21 cells and incubated for 1 h. Cells were washed to remove the inoculum and replaced with fresh media (Figure 3A). This assay revealed a very strong significant virucidal activity of RcP resulting in the complete blockage of virus infection (*p* < 0.01) (Figure 3A).

Next, the effect of RcP on virus attachment was analyzed. For this, cells were incubated with virus in the presence of RcP at 4°C for 1 h; upon these conditions, virus is able to attach to cell membrane receptor, but not to enter the host cells. Then, cells were washed and fresh media was added (Figure 3B). Data obtained from this assay showed that RcP reduced 90% of virus replication (*p* < 0.01), indicating that RcP reduced virus binding to the host cells (Figure 3B). Finally, the experiment was repeated allowing incubation with virus and compound for extra 30 min at 37°C after the attachment at 4°C. This assay was designed to include entry and uncoating steps (Tumkosit et al., 2019). Cells were washed and a fresh media was added (Figure 3C). The results demonstrated that under this protocol of treatment, the complex inhibited up to 55% of the viral infectivity (*p* < 0.01) (Figure 3C). Collectively, these data demonstrated that RcP was able to abrogate the early stages of virus entry to the host cells (Figure 3). The strongest effect was observed in virucidal experiments; effects on other stages of infection may be consequences of this activities. This suggests that an anti-CHIKV mechanism of action for this complex might be related to a direct action on the virus structure, resulting in the virions inactivation and/or inability to bind to cellular receptors.

### Potential Mechanisms of Action of RcP

**FIGURE 4:**
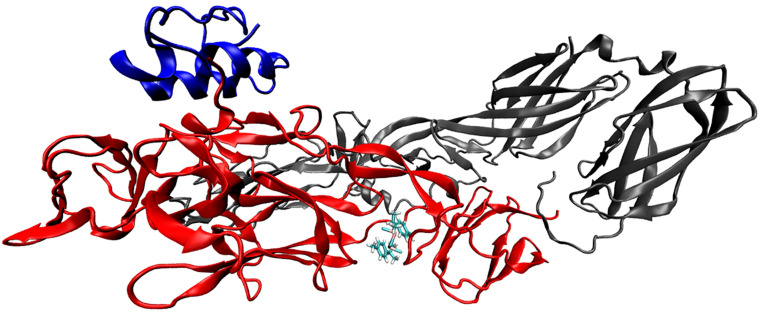
The CHIKV glycoprotein complexed with RcP. The E3 envelope (chain A) is presented in blue, the E2 envelope (chain B) in red and the E1 envelope (chain F) in gray. The NewCartoon representation was used for the glycoprotein and the licorice representation used for the ligand.

**FIGURE 5:**
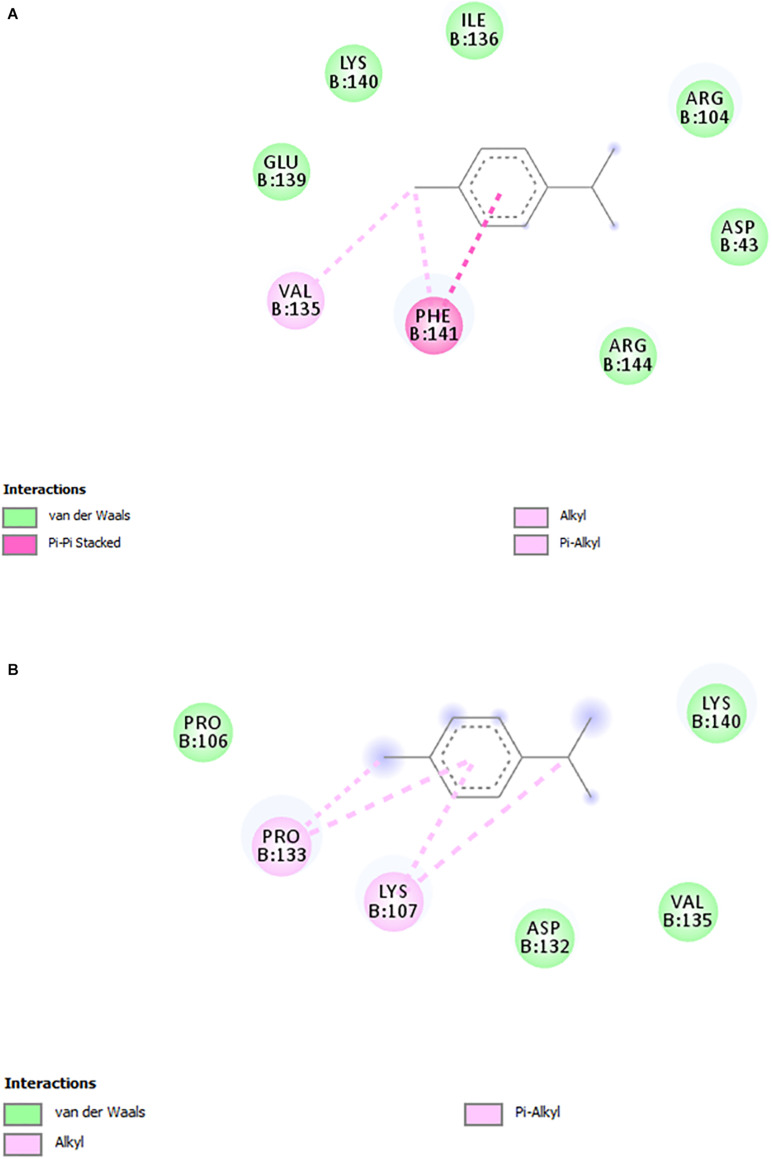
2D interactions diagrams (DS Visualizer) between the loop residues of the E2 glycoprotein and RcP. **(A)** Interactions between E2 envelope glycoprotein and ring 1. **(B)** Interactions between E2 envelope glycoprotein and ring 2. Non-polar hydrogen atoms were omitted for clarity.

Based on the results that showed RcP interfering with CHIKV entry to the host cells, molecular docking calculations were performed in order to investigate possible binding mode and interactions between RcP and CHIKV glycoproteins. The *in silico* analysis demonstrated that RcP was predicted to dock between two loops of the chain B of the E2 envelope glycoprotein (Figure 4), and the highest docking score presented a binding energy of −7.68 kcal mol^–1^ and a inhibition constant (K_*i*_) of 2.35 μM, showing that this compound might interact with CHIKV E2 (Chain B). The molecular docking study also suggested that RcP showed different interactions between its aromatic rings and the loop of amino acids of the E2 glycoprotein, mainly through hydrophobic interactions and one pi-pi stacking (Figure 5). However, the ruthenium and the chloride atoms did not participate in the principal docking pose, which accessed 9 amino acids (GLU139, LYS140, ILE136, ARG104, ASP43, ARG144, PRO106, VAL135, and ASP132) through these hydrophobic interactions (Figure 5). The amino acid LYS140 hydrophobically interacted with the two α-Ph ligands through its methyl group (Figure 5). The pi-pi stacking only occurred with one aromatic ring of the α-Ph and the PHE141 amino acid (Figure 5A). Therefore, the ruthenium—phenyl interaction was of fundamental importance for complex stabilization and, consequently, the simultaneous interaction between the two alpha-phellandrene with the two loops of chain B.

**FIGURE 6:**
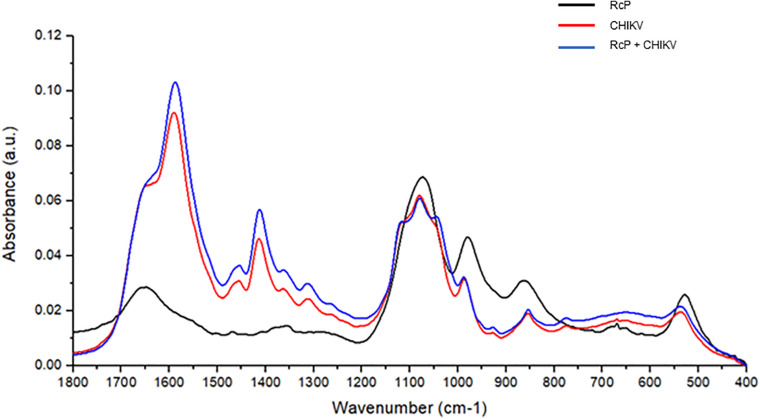
Representative infrared average spectrum of RcP. CHIKV virions, RcP and RcP + CHIKV virions, which contains several molecular functional groups in lipids, proteins, glycoproteins and nucleic acid.

**FIGURE 7:**
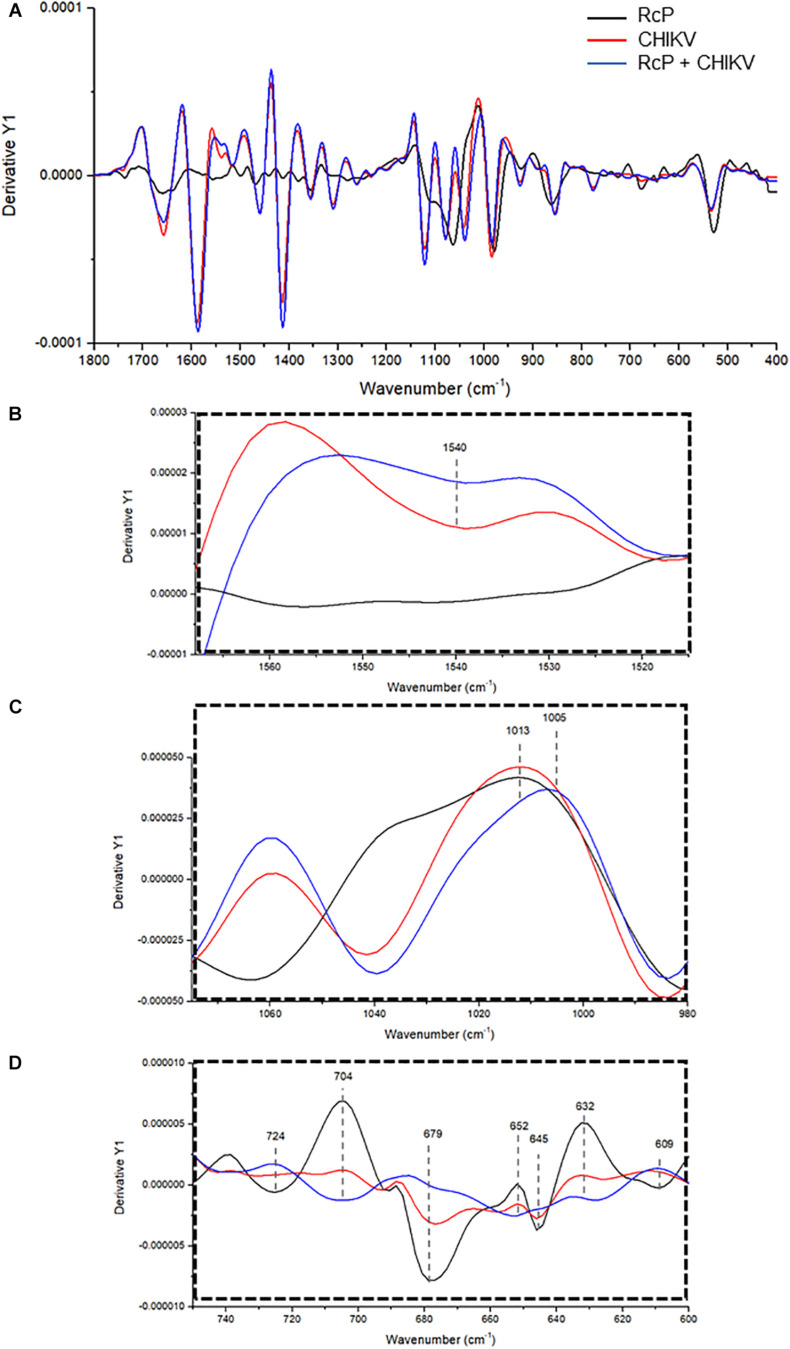
Representative infrared average spectrum of second derivative. **(A)** Analysis from CHIKV virion, RcP and RcP + CHIKV virion. **(B–D)** Second derivative analysis, which the value heights indicate the intensity of each functional group.

To further investigate the interaction between RcP and CHIKV virions, ATR-FTIR spectroscopy spectral analysis was performed. The vibrational modes detected in CHIKV virion and/or RcP are shown in Table 1. The representative infrared spectra of RcP, CHIKV virions, or RcP plus CHIKV virions, which contains different biochemical functional groups such as lipids, proteins, glycoproteins and nucleic acid, are represented in Figure 6. We were particularly interested in confirming the interaction of RcP with CHIKV virions which can be represented by the molecular infrared analysis. The representative infrared spectra of the second derivative analysis of RcP, CHIKV virions, or RcP plus CHIKV virions are displayed in Figure 7A. In the second derivative analysis, which the value heights indicated the intensity of each functional group, a reduction in intensity of Amide II [ν (N–H), ν (C–N)] at 1,540 cm^–1^ in RcP plus CHIKV virions suggests interaction of RcP with proteins of CHIKV virions (Figure 7B). The binding interaction was also revealed by spectral shifting of the 1,013–1,005 cm^–1^, which indicates interaction with vs. (CO-O-C) presents in glycoprotein derived from RcP and/or CHIKV virions (Figure 7C). Additionally, it was observed the decrease in intensity of 724, 679, 645, and 609 cm^–1^ in RcP plus CHIKV, which indicates interaction of C-H rocking of CH_2_ and S-O bending, reinforcing interaction. The binding interaction was additionally confirmed by the increase in intensity of 704, 652, and 632 cm^–1^ in RcP plus CHIKV virions, which indicates formation in the presence of OH out-of-plane bend (Figure 7D).

**TABLE 1:** Identification of functional group and molecular source of respective vibrational modes in ruthenium α-phellandrene complex (RcP) plus CHIKV sample.

Vibrational mode (cm^1^)	Proposed functional group	Molecular source
1,540	Amide II [ν (N–H), ν (C–N)]	Protein
1,013	vs. (CO-O-C)	Glycoprotein/Carbohydrates
1,005	vs. (CO-O-C)	Glycoprotein/Carbohydrates
724 704 679 652 645 632 609	C-H rocking of CH2 Unassigned band S-O bending OH out-of-plane bend Unassigned band OH out-of-plane bend S-O bending	Fatty acids, non-specific proteins Sulfates components Protein and lipids Non-specific Protein, Lipids Sulfates components

**Assignments of main wavenumbers of sample ATR-FTIR spectra. ν, stretching vibrations; δ, bending vibrations; s, symmetric vibrations and as, asymmetric vibrations.**

## Discussion

Chikungunya virus (CHIKV) has recently attracted worldwide public health attention due to massive outbreaks (Gould et al., 2017) and its ability to cause highly debilitating chronic disease that may persist for months or even years (Cunha and Trinta, 2017). CHIKV was first described in the 1950s (Robinsson, 1955), however, there are no approved therapeutics or vaccines against this infection (Mathew et al., 2017; Stegmann-Planchard et al., 2019). Thus, the search for new molecules with anti-CHIKV activity is critical in this public health issue.

In this study, the anti-CHIKV activity of the ruthenium and alpha-phellandrene complex (RcP) was investigated. For years, RcP has been showing potential activity against cancer (Dougan and Sadler, 2007; Dyson, 2007). However, its activity against viruses remains unknown.

Our results showed that RcP significantly reduced virus entry to host cells at non-toxic concentrations, and therefore, the effect of this complex was further evaluated on the early stages of CHIKV infection. RcP demonstrated a moderate yet significant activity on the virus uncoating and strong action on inhibiting virus attachment or as a virucide, being the latest the strongest activity of RcP. Several studies demonstrated the biological activities of this complex (Habtemariam et al., 2006; Savić et al., 2020), however, to the best of our knowledge there is no description of its antiviral activity.

The virucidal effect was the strongest activity observed for RcP which might suggest that an anti-CHIKV mechanism of action for this complex might be related to a direct action on the viral particle envelope. Previous studies have shown that the virucidal effect of several compounds is closely related to an action on the virus envelope (Tang et al., 1990; Schuhmacher et al., 2003; Carravilla et al., 2017; Kong et al., 2019; Russo et al., 2019). This suggests the existence of interactions between CHIKV envelope glycoproteins and RcP. Based on these data, molecular coupling calculations were performed to investigate the possible binding mode and interactions between RcP and CHIKV glycoproteins. Our results indicated that RcP can bind to a location between two loops of the E2 envelope glycoprotein B chain. Studies have already shown that the glycoprotein E2 is responsible for the binding of the virus to the cell receptor (Moller-tank et al., 2013; Fongsaran et al., 2014; Silva et al., 2014). Rashad and Keller (2013) showed that when small molecules bind to a region between the two loops of the E2 B chain, the movement of the glycoprotein domains can be modified and prevent the virus from entering the cell (Rashad and Keller, 2013). We suggest that RcP may be binding to such a site and preventing the virus from binding to the cell. Furthermore, through molecular interactions observed by infrared spectroscopy, we can suggest that RcP may interfere with CHIKV glycoprotein and lipid sites, corroborating that there is an interaction between the viral envelope and RcP.

FTIR spectroscopy has been used for detection and quantification of viral pathogens, and could be also required for molecular responses to viral infections in tissue, cell or biofluids (Lee-Montiel et al., 2011). Recently, this tool was also used to identify molecular structures in viral components under changes in pH and to investigate molecular modification of viruses in the presence of antiviral compounds (Lai and Freed, 2015; Gamage et al., 2018). In this context, the interactions between RcP and CHIKV virions could be suggested based on changes observed in intensity of vibrational mode at 1,540 cm^–1^, which indicate the association of RcP with Amide II functional groups present in viral proteins. The shift in a specific vibrational mode can also indicates interaction between molecules (Popova and Hincha, 2003). It was revealed in our results by infrared spectral shifting of the 1,013–1,005 cm^–1^, indicating an interaction of CO-O-C presents in glycoprotein of CHIKV virions. Therefore, the assumption of RcP interaction with the amino acids of the CHIKV glycoprotein E2 by the molecular docking analysis could be reinforced by infrared spectroscopy analysis. The reduction in the intensity of 724, 679, 645, and 609 cm^–1^ in RcP plus CHIKV virions compared with only RcP suggests interaction in C-H rocking and S-O bending present in RcP. Thus, ATR-FTIR represented a powerful, sustainable, fast, cost-effective and label-free platform to the understanding of the interaction of CHIKV glycoproteins with RcP and, consequently, providing insights on the mode of action of this organometallic complex.

In summary, we showed that RcP was able to strongly inhibit CHIKV infectivity, acting on the early stages of virus infection, mainly as a virucidal inhibitor. To the best of our knowledge, this is the first description of the antiviral activity of RcP against CHIKV. This data may be useful for the development of future antivirals against CHIKV that will provide a relevant advance to the public health in the combat of Chikungunya fever.

## Data Availability Statement

The original contributions presented in the study are included in the article/supplementary materials, further inquiries can be directed to the corresponding author/s.

## Author Contributions

DO, IS, DM, YG, LC-S, EF, and AJ: acquisition of data and results analysis. DO: drafting of the manuscript. RS-S, GP, EF, NN-J, CP, AM, MH, and AJ: study design, supervision, and critical revision. All authors reviewed the manuscript.

## Conflict of Interest

The authors declare that the research was conducted in the absence of any commercial or financial relationships that could be construed as a potential conflict of interest.
